# Experimental Investigation and Thermodynamic Modeling of Influence of Nickel and Titanium Content on the Structure and Selected Properties of Tin Bronzes

**DOI:** 10.3390/ma14205944

**Published:** 2021-10-10

**Authors:** Janusz Kozana, Aldona Garbacz-Klempka, Marcin Piękoś, Małgorzata Perek-Nowak, Paweł Pałka

**Affiliations:** 1 Faculty of Foundry Engineering, AGH University of Science and Technology, Reymonta 23, 30-059 Krakow, Poland; jkozana@agh.edu.pl (J.K.); mpiekos@agh.edu.pl (M.P.); 2 Faculty of Non-Ferrous Metals, AGH University of Science and Technology, Mickiewicza 30, 30-059 Krakow, Poland; mperek@agh.edu.pl (M.P.-N.); pawel.palka@agh.edu.pl (P.P.)

**Keywords:** copper alloys, Cu-Sn bronze alloys, solidification process, mechanical properties, metallography, CALPHAD

## Abstract

Investigations are conducted in order to maintain or to improve the selected properties of the group of foundry copper-tin alloys with nickel and titanium additions, at a limited fraction of the critical (deficit) element such as tin. The crystallisation process, as well as changes of the microstructure and selected mechanical properties of the CuSn8 alloy—occurring due to introducing alloying additions—were analysed. Investigations of the macro and microstructure were performed using optical and scanning electron microscopy. Based on the thermal analysis and thermodynamic modelling using the CALPHAD (CALculations of PHAse Diagrams) method, the crystallisation process was analysed. The identification of phases was performed by XRD (X-ray diffraction). In addition, such parameters as tensile strength-UTS, elongation-A and hardness-HBS were tested. Under the influence of the introduced titanium, the columnar crystals are reduced due to the crystallisation of the alloy at the walls of the mould. Precipitations (intermetallic phases) crystallize first (primary). The intermetallic phases associated with the presence of the alloying elements nickel and titanium are located in the interdendritic regions. In tin bronzes with titanium additions, hardness (HBS) increases, tensile strength (UTS) negligibly decreases, while elongation (A) significantly decreases. In the case of CuSnNi bronze, the addition of 0.2 wt.% Ti increases the hardness and increases ultimate tensile strength (UTS), while reducing the elongation (A). Higher Ti additions increase HBS, slightly decrease the tensile strength, and significantly reduce the elongation.

## 1. Introduction

Copper alloys are the most known and researched alloys [1,2]. Tin bronzes, known and applied already in the antiquity, still constitute the important and irreplaceable group of alloys used for production of machines and parts of devices [3,4,5,6,7,8,9]. Advantages of these alloys are, among others, good flowability, strength, ductility, high corrosion resistance and also abrasion resistance at high loads [10,11,12]. In Cu–Sn alloys, Sn content is important since it influences its strength and functional properties [13,14,15,16,17,18]. Due to higher requirements to modern materials, high prices of tin and its short supply, the substitutes of this group of elements are looked for and applied [19,20,21,22]. This is realised by the development of new alloys based on alloying additions, such as: zinc, lead, phosphor, nickel, iron, titanium and aluminium, as well as on refining, modification and the way of casting (gravity, centrifugal, sand or metal moulds) [23,24,25,26,27,28,29].

The authors have already carried out research concerning various alloy additions influencing the properties of bronzes [23,30,31,32,33]. Regardless of the fact that several multi-component bronzes based on the Cu-Sn composition were developed and described in the scientific literature, looking for new alloys and an in-depth analysing of the results is fully justified [34,35,36]. This allows for modifying alloys from the Cu-Sn system and widening their application range [37,38,39,40,41,42]. Cast CuSn alloys are usually alloys containing 10 and more in wt.% Sn. Alloys with a lower Sn content, such as CuSn4, CuSn6, CuSn8, are used, but as alloys used for the production of ingots using continuous casting, then by plastic working they are formed into sections of different cross-sections. Therefore, the development of casting alloys with a tin content of less than 10 wt.% can be defined as tin-reduced casting materials.

The main aim of performed studies was the optimisation of alloys based on the Cu-Sn system by means of alloying additions, i.e., nickel and titanium at a decreased tin content (8 wt.%). It was assumed that introductions of alloying additions would influence, maintain, and even improve tin’s bronzes mechanical properties—primarily increasing its hardness (HBS) and ultimate tensile strength (UTS) at a determined ductility [32,43,44].

## 2. Materials and Methods

The investigations concerning changes caused by titanium additions to tin bronzes were realised in the Laboratory of Non-Ferrous Metals Casting of the Faculty of Foundry Engineering of AGH–University of Science and Technology, Krakow. The influence of titanium additions was analysed with using two base alloys: CuSn8 and CuSn8Ni3. The additives Ni and Ti were selected on the basis of their own experimental research, so as to show the effect at different contents. The selection of the amount of additives was also preceded by an analysis of phase diagrams [1,2].

### 2.1. Preparation of Alloys

Investigations of properties of tin and tin-nickel bronzes were realised by the application of a metal charge prepared from the CuSn10 bronze (acc. EN 1982, PN-91/H87026) (CuSn10-Hutmen LLC, Katowice, Poland), electrolytic nickel and cathodic copper, Cu-ETP (EN 1652) (nickel and copper- KGHM Polska Miedź S.A., Lubin, Poland). The Ti addition was introduced into a metal bath at a temperature of 1150–1200 °C. Melts were carried out in the inductive furnace of a medium frequency, in the chamotte-graphite crucible. Bronze CuSn10 was added in the form of cast billets, nickel and cathode copper in the form of cut pieces of cathodes, and titanium in the form of pieces of sheet metal 0.5 mm thick. The entire metal charge was approximately 8 kg. Producer of charge materials: CuSn10-Hutmen LLC, nickel and copper-KGHM Polska Miedź S.A.

While performing melts, protective coverings of charcoal were applied on surfaces of a charge and metal bath. Applying charcoal to the surface of a liquid copper alloy reduces the oxygen contamination of the alloy. A deoxidising addition in the form of phosphorous copper CuP8 (0.03 wt.%) (Hutmen LLC, Katowice, Poland) was also introduced to take care of the stable and reproducible conditions of the melt’s production. The melts were poured to metal moulds for solidification. Eight alloys of various chemical compositions, different contents of tin, nickel, titanium and copper, were realised. The remaining metal elements were treated as contaminations.

The chemical compositions of alloys obtained according to the planned experiment were determined by the X-ray fluorescence spectrometer with the energy dispersion (SPECTRO MIDEX, Kleve, Germany). Results of the tested chemical composition of individual alloys are given in Table 1.

### 2.2. Microstructure Analysis and Phase Analysis

Metallographic macro- and microstructure observations were carried out by the optical microscopy (NIKON EclipseLV150, Tokyo, Japan) and scanning electron microscopy (Hitachi S-3400N, Tokyo, Japan). In addition, elemental analysis in microregions by energy dispersive X-ray spectroscopy (NORAN System SIX by Thermo Fisher Scientific, Thermo Electron Scientific Instruments LLC, Madison, WI, USA) was made. The phase identification was carried out by the X-ray powder diffraction method in Bruker D-8 Discover Advance diffractometer (Bruker D8 Advance, Karlsruhe, Germany) with a Cu tube. The X-ray powder diffraction scan was made in a range 2θ of 20–120° with a step of 0.02°. The results of powder diffraction are presented for samples with the maximum concentration of alloying elements, since they were the most distinct and characteristic for the analysed group of alloys.

### 2.3. Mechanical Properties Analysis

The tensile strength UTS and elongation A (number of tests—5), were determined by LabTest ZD20 Labor Tech (V = 10 MPa/s), while Brinell hardness HBS 2.5/62.5 (number of tests-3) measurements were made using the HPO-250 hardness tester (WPM, Labor Tech, Leipzig, Germany).

### 2.4. Thermal-Derivative TDA Analysis Method

With the use of the thermal-derivative (TDA) analysis, the characteristic thermal effects resulting from phase changes occurring during the crystallisation of the tested Cu-Sn alloys were recorded and determined. For the thermal analysis, the mantle K-type thermoelement (diameter 0.5 mm) was used. Temperature measurements were made by the laboratory multimeter Keysight 34972A (Santa Rosa, CA, USA), equipped with a 16-channel Reed Multiplexer Module 34902A.

### 2.5. Thermodynamic Modelling

Ready alloys were cast into the metal mould in order to obtain castings for further research. On the bases of the TDA curves and thermodynamic modelling using the CALPHAD (CALculation of PHAse Diagrams) software with a packet Thermo-Calc for copper alloys TCCU:TCS Cu-based Alloys Database, investigations of the phase analysis were also made.

## 3. Results

On the basis of TDA results, temperatures of characteristic changes were determined. The most noticeable influence of alloying elements is observed at the beginning of the crystallisation of the examined alloys. Addition of 3 wt.% Ni makes the melting point to increase by 20 °C in relation to the alloy without this additive. The increasing proportion of titanium addition in both cases successively lowers the melting point. The temperatures T2, T3 are characteristic for the formation of chemical composition-dependent intermetallic phases during crystallisation. The disclosed T4 temperature, taken as the solidus temperature, fluctuates in the range of 751–773 °C for most of the tested alloys, with the exception of the CuSn8Ti2 alloy for which this temperature was 860 °C. Values of temperatures T1–T4 are shown in Table 2.

**Figure 1:**
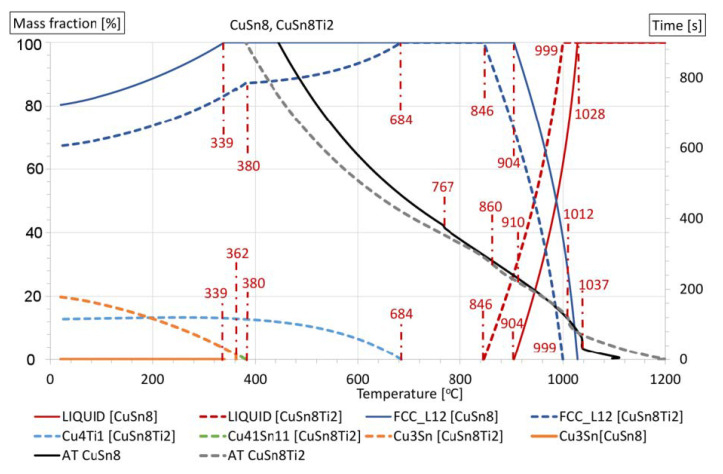
Crystallisation pathways of selected alloys: CuSn8 and CuSn8Ti2, on the bases of experimental data and thermodynamic modelling by the CALPHAD method and AT.

Changes occurring in the thermal analysis diagrams allow for observing different solidification character of CuSn8 and CuSn8Ni3 alloys with titanium additions. In the CuSn8Ti2 alloy, the additional, newly formed phases, especially the Cu4Ti1 phase, are the result of CALPHAD calculation (Figure 1).

Both thermal analyses: experimental and the one determined on the basis of thermodynamic modelling by the CALPHAD method, allowed for presenting the crystallisation process of tested alloys (Figure 1). The determined, phase transformation temperatures occurring at a solidification and cooling of alloys are listed in Table 3. The graph also shows the experimental crystallisation curves (AT of CuSn8, AT of CuSn8Ti2). The experimental curves in Figure 1 represent the alloys with the designations AT CuSn8 and AT CuSnTi2, respectively. The remaining curves are the result of CALPHAD modelling. CALPHAD modelling assumes an equilibrium course of the crystallisation process, hence the obtained results of the thermal-derivative analysis do not exactly match. The greatest similarity can be observed in the case of the liquidus temperature T1, and the differences are in the range of 9–14 °C. In the case of the T1 temperature (liquidus temperature), we observe the appearance of the first crystals (solid Cu(Sn) solution). At Figure 1, solid line curves refer to the CuSn8 alloy, and dashed line curves to CuSnTi2 alloy. In addition, in the diagram legend, for each phase, the appropriate stop is described in parentheses. As the metal temperature drops to the T2 value, the LIQUID (solidus temperature) disappears, and the proportion of the solid solution increases. Below the temperature T2, transformations already take place in the solid state, and phases form depending on the chemical composition of the alloys tested. For the presented CALPHAD modelling results for the CuSn8 alloy, due to the temperature dropping to the T6 value, the Cu3Sn phase crystallizes (at the expense of the depletion of the Cu(Sn) solid solution). In the remaining alloys, as a result of the presence of additional alloying elements (Ni, Ti) in the CuSn8 alloy, the following phases will crystallize:

− CuSn8Ti2: T3-Cu4Ti1, T5-Cu41Sn11, T6-Cu3Sn,
− CuSn8Ni3: T3-CuSn_GAMMA, T4-Cu10Sn3, T5-Cu6Sn5_HT, T6-Cu3Sn,
− CuSn8Ni3Ti2: T3-CuSn_GAMMA, Cu4Ti1, T4-Cu10Sn3, T5-Cu6Sn5_HT, T6-Cu3Sn.

**Figure 2:**
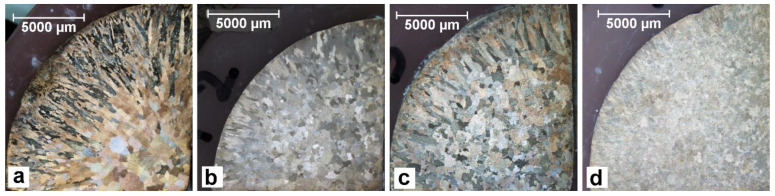
Microstructure of the alloy, 0.67x, metal mould: (**a**) CuSn8; (**b**) CuSn8Ti2; (**c**) CuSn8Ni3; (**d**) CuSn8NiTi2 [32].

The macrostructure images also indicate the changing nature of the solidifying of CuSn8 alloys (Figure 2). The grains shape and their fractions (grain size) on the cross-section of the sample-ingot are varying.

**Figure 3:**
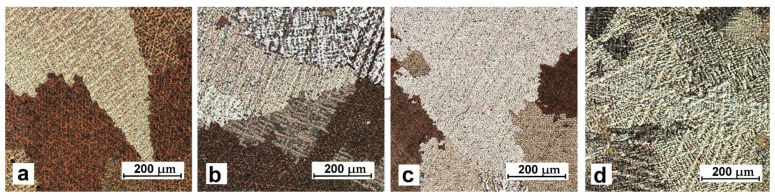
Microstructure of the alloy, metal mould: (**a**) CuSn8; (**b**) CuSn8Ti0.2; (**c**) CuSn8Ti0.7; (**d**) CuSn8Ti2.

**Figure 4:**
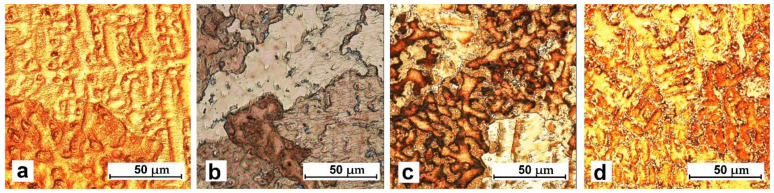
Microstructure of the alloy, metal mould: (**a**) CuSn8Ni3; (**b**) CuSn8Ni3Ti0.2; (**c**) CuSn8Ni3Ti0.4; (**d**) CuSn8Ni3Ti2.

The titanium addition in CuSn8 and CuSn8Ni3 alloys causes narrowing of the skin zone and a significant refinement of equiaxial grains in the central part of the sample. The observed changes result from the influence of titanium in terms of crystallisation (creation of additional nuclei and grain refinement). However, this scope of changes requires further detailed research. The influence of the titanium addition also causes changes in the microstructure images (Figure 3 and Figure 4). These changes will be discussed later in the article.

The precipitates related to the presence of alloying additions of nickel and titanium are located in interdendritic regions.

**Figure 5:**
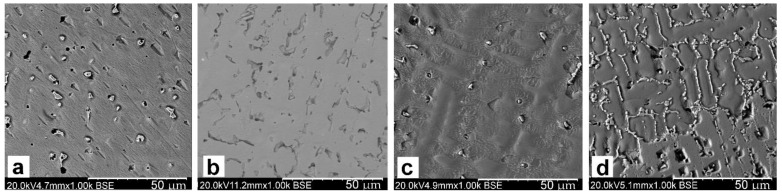
The SEM image of the alloy surface, metal mould: (**a**) CuSn8; (**b**) CuSn8Ti2; (**c**) CuSn8Ni3; (**d**) CuSn8Ni3Ti2.

**Figure 6:**
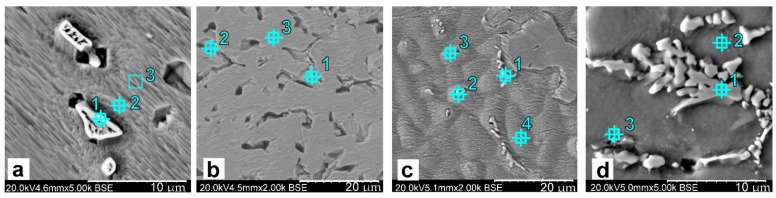
The SEM-EDS image of the alloy surface, metal mould: (**a**) CuSn8; (**b**) CuSn8Ti2; (**c**) CuSn8Ni3Ti0.2; (**d**) CuSn8Ni3Ti2.

**Figure 7:**
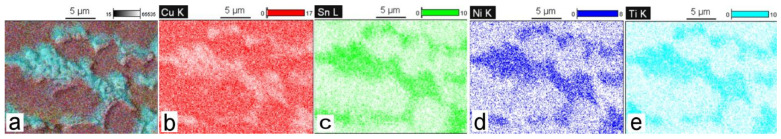
Element distribution maps for CuSn8Ni3Ti2 alloy, with separation of the intermetallic phase: (**a**) maps of elements; (**b**) Cu; (**c**) Sn; (**d**) Ni; (**e**) Ti.

The selected samples of analysed alloys were subjected to investigations by means of the scanning electron microscopy. SEM images of the surfaces of investigated samples are shown in Figure 5, Figure 6 and Figure 7. Precipitates of eutectoid and intermetallic phases occur in interdendritic spaces (Cu_6_Sn_5_, Ti_2_Sn, CuSn_3_Ti_5_, Ni_2_SnTi, NiSnTi, Cu_6_Sn_5_).

The performed analyses of SEM-EDS (Figure 6, Table 4, Table 5, Table 6 and Table 7) confirmed multiphase structures of discussed alloys. Introduced additions of nickel and titanium cause crystallisations of multicomponent phases containing copper, tin, nickel and also titanium. It was found that tin occurred in the solid solution within a range of 2–14%, while in phases at a level of 30 wt.%. The introduced nickel addition occurs in the composition (3.5 wt.%), as well as in intermetallic phases within a range of 8–25 wt.% (on the average: 15 wt.%). Titanium in CuSn8 alloys occurs in the solid solution at a level of 0.7 wt.%, while the Ti excess occurs in phases and linearly increases when the Ti fraction increases from 7 to 25 wt.%. Titanium in CuSn8Ni alloys occurs mainly in intermetallic phases (on the average: 12 wt.%), and small amounts of Ti also occur in the solid solution (0–1.2 wt.%).

**Figure 8:**
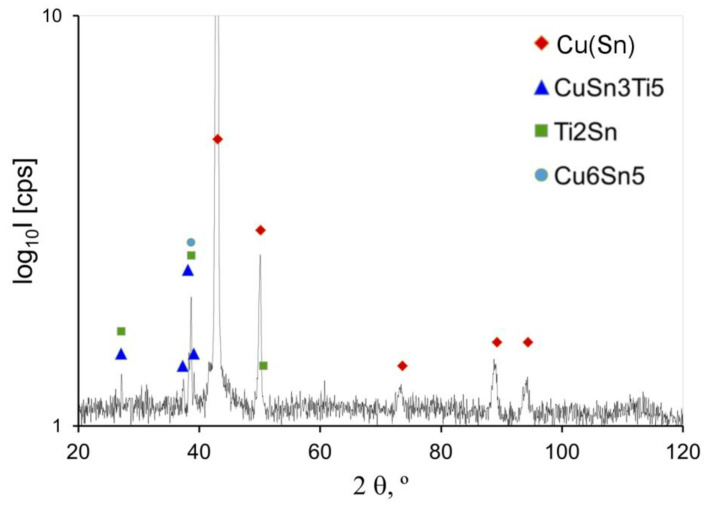
Powder diffraction spectrum of the CuSn8Ti2 alloy.

**Figure 9:**
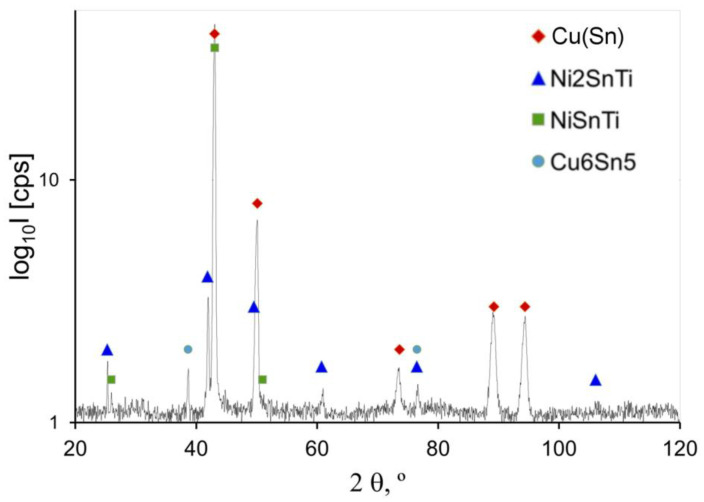
Powder diffraction spectrum of the CuSn8Ni3Ti2 alloy.

The X-ray diffraction tests (XRD) allowed for identifying the crystallised phases, while, on the basis of their correlation with the thermodynamic modelling by the CALPHAD method, it was possible to determine their amounts. The results of analyses for the selected alloys are presented in Figure 8 and Figure 9.

Crystallised intermetallic phases of variable volumetric fractions in the CuSn8Ti2 alloy are: Cu_6_Sn_5,_ Ti_2_Sn, and CuSn_3_Ti_5_. The phase analysis for CuSn8Ni3Ti2 alloy was also indicated at various fractions of intermetallic phases Ni_2_SnTi, NiSnTi and Cu_6_Sn_5_ (Figure 9).

**Figure 10:**
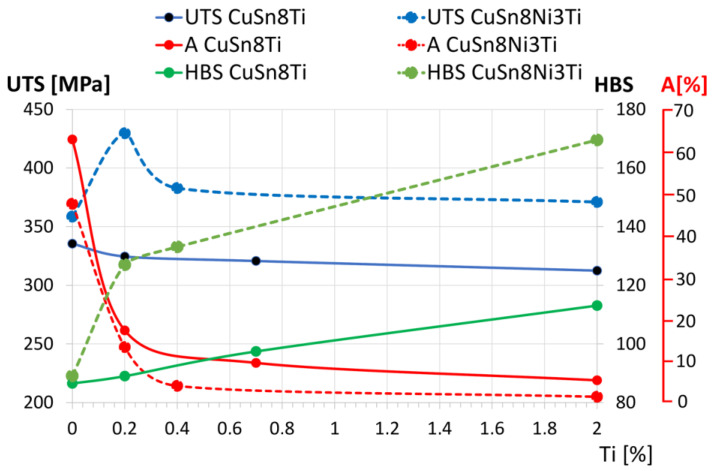
Graphical representation of the mechanical properties of samples made of CuSn8+Ti and CuSn8Ni3+Ti alloys.

The results of mechanical tests i.e., tensile strength (UTS), elongation (A) and hardness (HBS) are presented in Figure 10 and Table 8.

The obtained results of mechanical properties (Figure 10, Table 8) indicate a significant improvement of tensile strength (UTS) and hardness (HBS), at a linear decreasing of the ductility. The addition of 0.2 wt.% of titanium to the CuSn8Ni3 alloy increases its hardness (HBS) by more than 40%, at a negligible decrease of its elongation (A). Increasing the Ti addition over 0.2 wt.% in the analysed alloys causes not only grain size reduction but also changes in the microstructure. The effect of this is an increase in hardness while reducing the tensile strength (UTS).

## 4. Discussion

The group of Cu-Sn alloys selected for tests as well as the obtained results caused by nickel and titanium additions indicated several interesting changes within crystallisation, optical and scanning metallography and identifications of precipitates and phases, which were tested by means of the scanning microscopy SEM-EDS and XRD.

The obtained alloys had variable chemical compositions with respect to their main components and also with respect to applied alloying additions. The thermal analysis of CuSn8Ti and CuSn8Ni3Ti alloys indicates changes of temperatures of individual, characteristic phase transformations. Nickel increases a temperature of the beginning of solidification T1, while the addition of titanium decreases T1 value, especially in alloys with nickel additions. Solidus temperature of the alloys, marked T4, also decreases when nickel and titanium are added. This decrease is more intensive in the case of alloys with nickel.

By introducing the addition of nickel to CuSn alloys, the solidification range of the stack alloy changes slightly; however, as a result of the increased crystallisation temperature, the casting temperature of these alloys should be higher. Modelling CALPHAD with the TC method allows the analysis of the alloy’s crystallisation process and helps in the TDA analysis of solidification of alloys under real casting crystallisation conditions. The slight differences in the results obtained with both methods are certainly caused by an imperfect adjustment of the crystallisation conditions for theoretical and real research. CALPHAD modelling measurably indicates the specific temperature values of the crystallizing phases and their chemical composition. According to TC (CALPHAD), the equilibrium modelling of the investigated alloys suggests the formation (in an ambient temperature) of the basic three phases: Cu_3_Sn, Cu_4_Ti and Cu(Sn) (which is mainly Cu). The chemical composition of Cu_4_Ti phase: 84.4% copper and 15.6% titanium, while the chemical composition of Cu_3_Sn phase: 61.7% Cu and 38.3% Sn. An introduction of nickel into this system causes changes in the composition of the Cu_3_Sn phase for the Cu_2_(NiSn) phase. The copper fraction falls to 46.6% and one of nickel increases to 15.5%, while the tin fraction remains the same as before (38.3%).

Investigations of macro-grains of alloy samples by means of optical microscopy indicated changes of these grains’ morphology within ingots. Columnar crystals occur from the side of a metal mould (ingot edge). Inside the ingot, equiaxial crystals can be seen. The introduced titanium narrows the zone where equiaxial crystals occur, especially in alloys with nickel. The nickel addition increases slightly the width of equiaxial crystals at a simultaneous refinement of these crystals in the central part of ingots.

Microstructural tests revealed the typical structure of CuSn group alloys: solid solution dendrities within grains with marked boundaries. Precipitates of different phases occur on grain boundaries and in dendritic zones.

The analysis of the SEM-EDS results indicates variable chemical composition of individual components of the structure. The elements’ distribution map also revealed the highest fraction of copper in matrix. Tin, nickel and titanium occur in the matrix and—in higher amounts—in intermetallic phases.

The results of XRD investigations allowed for finding out intermetallic phases of various configurations in the alloys structure from the systems: Cu-Sn-Ti, Ti-Sn, Cu-Sn and Ni-Sn-Ti.

The obtained results of OM and SEM confirmed the increased share of intermetallic phases with the introduced addition of titanium, concentrated mainly in the interdendritic spaces, which, together with the addition of nickel, are responsible for the increase in hardness.

The summary of the observed changes, concerning solidifications of analysed alloys, constitutes the obtained results of the selected mechanical properties.

The mechanical properties present an increase in tensile strength (UTS) and hardness (HBS) and a considerable decrease in ductility. However, the addition of only 0.2 wt.% of Ti to CuSn8Ni3 bronze causes a significant increase in the hardness (over 40%) and a negligible decrease of elongation. The presented studies, apart from providing the cognitive knowledge, indicated the possibility of improving the selected properties.

The titanium introduced to the CuSn8 and CuSn8Ni3 alloys affects both the macrograin shaping process and changes the internal structure in the terms of microstructure. The macro changes were confirmed in metallographic studies using a stereoscopic microscope. The images of the surface layer of the solidified ingots in the metal form presented in Figure 2a–d showed changes in the edge zone, solidified in conditions of faster solidification (a decrease in the columnar crystal zone).

In the edge zone (near the wall of the solidifying ingot mould), one can observe the crystals in the form of elongated lumps arranged along the radius of the ingot. Comparing the surface pictures of the ingots made of CuSn8 alloys (Figure 2a) and CuSn8Ni3 (Figure 2c), no significant differences can be seen in the recorded images. Only the width of the pillar crystals is slightly smaller for the alloy without the addition of Ni. The Ti introduced into the CuSn8 alloy changes the edge zone, shortening the length of the columnar crystals.

The presence of Ti in the CuSn8Ni3 alloy causes more visible changes. The edge zone (bar crystals) is reduced from about 5000 µm to about 2000 µm.

It is known that the casting first solidifies at the wall of the metal mould towards the center of the casting (directional solidification), and then the rest of the casting solidifies more evenly (volumetric solidification).

Since the introduced titanium reduces the directional solidification area, it means that the presence of titanium in the analysed alloy to some extent leads to the formation of additional nuclei, which contribute to faster solidification of the bulk casting, reducing the effect of faster solidification of the casting from the wall of the metal mould.

Obtaining this change in solidification conditions can lead to a more uniform structure of the casting. An additional benefit from the use of titanium was revealed in the conducted research (ingot macrostructure) in terms of the reduction of the macrosized grain size in the equiaxial crystal zone (center of the casting, beyond the edge zone). The grain refinement should also be attributed to the role of a certain part of the titanium in terms of generating additional nucleating agents.

However, this area of research requires further experience and analysis.

The presence of titanium also induces changes in the image of the microstructure (using optical microscopy in Figure 3 and Figure 4 and scanning electron microscopy in Figure 5).

The presence of titanium in the CuSn8 and CuSn8Ni3 alloys leads to the formation of intermetallic phases, located mainly at the grain boundaries and in interdendritic spaces.

The studies with the use of SEM scanning microscopy (Figure 5 and Figure 6) and SEM-EDS (Figure 6) allowed for determining the type of crystallised phases in terms of the chemical composition of these precipitates. X-ray diffraction XRD studies and CALPHAD thermodynamic modelling showed the types of phases occurring against the background of tin solution in copper (Cu_6_Sn_5_, Ti_2_Sn, CuSn_3_Ti_5_, Ni_2_SnTi, NiSnTi, Cu_6_Sn_5_). However, after an in-depth analysis of the created intermetallic phases, the influence of the titanium in the formation of the nuclei for crystallisation requires in-depth research and analysis (which will certainly be the subject of further research).

The introduced Ti additives show a variable effect on mechanical properties depending on the proportion of the alloy additive used and the type of alloy (with or without nickel).

A small amount of Ti (0.2 wt.%) in the CuSn8 alloy slightly reduces UTS and increases HBS while at the same time significantly reducing A. This is due to the grain refining effect of titanium. This amount of titanium can also favor the formation of small size intermetallic phases. These changes cause a significant reduction in ductility. Increasing the addition of Ti favors the formation of more intermetallic phases which result in an increase in hardness.

In the case of the CuSn8Ni3 alloy, the presence of nickel with a small addition of Ti (0.2 wt.%) increases the tensile strength (maximum UTS value 420 MPa) and significantly increases the hardness (127 HBS) while reducing ductility (13.3%).

Increased titanium additions up to 0.4 wt.% and more lead to the formation of intermetallic phases, which reduce ductility, increase hardness, and make the alloy brittle; thus, the strength of UTS does not increase, or even slightly decreases. Conducting additional tests with the use of Ti additives may show that the extreme strength with optimal A and HBS parameters for the CuSn8 alloy occurs at the Ti content in the range of 0 to 0.2 wt.%.

## 5. Conclusions

The performed investigations concerning influences of nickel and titanium additions to CuSn8 tin bronze allowed for observing several changes within the crystallisation, macro- and microstructures, SEM-EDS, XRD results and selected mechanical properties. Experimental data were supplemented by the results of thermodynamic modelling performed by means of the CALPHAD method. The results of investigations were correlated and verified.

Based on the research, the following conclusions can be drawn:

1. The titanium addition to CuSn8 and CuSn8Ni3 alloys causes the narrowing of the edge zone (directional crystallisation) and a significant refinement of equiaxial grains in the central part of the sample.
2. Titanium additions show more intense influence on the change of HBS, A and UTS. The addition of Ti in the amount of 0.2 wt.% increases the hardness (HBS) from 89 to 127, increases ultimate tensile strength (UTS) from 358 MPa to 429 MPa, while it reduces the elongation (A) from 63% to 13%. Higher Ti additions increase HBS and stabilize UTS at the level of 370–380 MPa while reducing the A elongation to the range of 1–4%.
3. The optimal content of Ti in tin bronze in a Ni system is 0.2%. With this content, Ti has a modifying effect: it refines the microstructure and hardens the alloy (increasing in HBS), increasing the tensile (UTS) at the expense of reducing the elongation (A).

## Figures and Tables

**Table 1:** Chemical composition (wt.%) of tin Cu-Sn alloys with additions of nickel and titanium.

Alloy	Chemical Composition wt.%				
	Ti	Ni	Sn	Zn	Cu
CuSn8	<0.030	0.12	8.00	0.212	bal.
CuSn8Ti0.2	0.173	0.14	8.06	0.185	bal.
CuSn8Ti0.7	0.710	0.14	7.96	0.199	bal.
CuSn8Ti2	1.978	0.12	7.98	0.183	bal.
CuSn8Ni3	<0.030	3.32	7.29	0.145	bal.
CuSn8Ni3Ti0.2	0.146	3.28	7.99	0.201	bal.
CuSn8Ni3Ti0.4	0.385	3.33	7.87	0.186	bal.
CuSn8Ni3Ti2	2.426	3.19	7.41	0.168	bal.

**Table 2:** The results of the characteristic temperatures determined on the basis of the TDA analysis.

Alloy	Temperature [°C]			
	T1	T2	T3	T4
CuSn8	1037	-	-	767
CuSn8Ti0.2	1025	-	-	754
CuSn8Ti0.7	1023	846	-	751
CuSn8Ti2	1012	910	875	860
CuSn8Ni3	1057	907	-	772
CuSn8Ni3Ti0.2	1051	919	-	773
CuSn8Ni3Ti0.7	1046	952	837	772
CuSn8Ni3Ti2	1028	966	826	751

**Table 3:** Characteristic temperatures of crystallisation processes during cooling of selected alloys: CuSn8 and CuSn8Ti2, on the bases of thermodynamic modelling by the CALPHAD method.

Sample/Phase	Temperature [°C]							Mass Fraction at 20 [°C] [%]
	T1	T2	T3	T4	T5	T6	T7	
**CuSn8**								
LIQUID	1028	905	-	-	-	-	-	-
Cu(Sn)	1028	905	-	-	-	339	20	80.4
Cu3Sn	-	-	-	-	-	339	20	19.6
**CuSn8Ti2**								
LIQUID	999	-	846	-	-	-	-	-
Cu(Sn)	999	-	846	684	380	-	20	67.3
Cu3Sn	-	-		-	-	362	20	19.8
Cu41Sn11	-	-	--	-	380	362	-	-
Cu4Ti1	-	-	-	684	-	-	20	12.9
**CuSn8Ni3**								
LIQUID	1043	919	-	-	-	-	-	-
Cu(Sn)	1043	919	640	448	438	325	20	79.4
CuSn_GAMMA	-	-	640	448	439	-	-	-
Cu10Sn3	-	-	-	448	442	325	-	-
Cu6Sn5_HT	-	-	-	-	442	396	-	-
Cu3Sn	-	-	-	-	-	325	20	20.6
**CuSn8Ni3Ti2**								
LIQUID	1015	876	-	-	-	-	-	-
Cu(Sn)	1015	876	674	453	443	-	20	66.5
CuSn_GAMMA	-	-	646	-	443	-	-	-
Cu10Sn3	-	-	-	453	-	325	-	-
Cu6Sn5_HT	-	-	-	-	443	397	-	-
Cu3Sn	-	-	-	-	-	325	20	20.6
Cu4Ti1	-	-	674	-	-	-	20	12.9

**Table 4:** Chemical composition EDS wt.% of CuSn8 alloy for the Figure 6a image.

Alloy	Chemical Composition wt.%	
	Cu	Sn
CuSn8_pt1	69.70	30.30
CuSn8_pt2	87.49	12.51
CuSn8_pt3	96.29	3.75

pt[numer], where [numer] = 1, 2, 3 indicates the measurement points.

**Table 5:** Chemical composition EDS wt.% of CuSn8Ti2 alloy for the Figure 6b image.

Alloy	Chemical Composition wt.%		
	Cu	Sn	Ti
CuSn8Ti2_pt1	38.79	32.60	28.61
CuSn8Ti2_pt2	50.01	26.83	23.16
CuSn8Ti2_pt3	97.44	1.71	0.85

pt[numer], where [numer] = 1, 2, 3 indicates the measurement points.

**Table 6:** Chemical composition EDS wt.% of CuSn8Ni3Ti0.2 alloy for the Figure 6c image.

Alloy	Chemical Composition wt.%			
	Cu	Sn	Ni	Ti
CuSn8Ni3Ti0.2_pt1	24.59	34.31	28.31	12.79
CuSn8Ni3Ti0.2_pt2	63.08	31.79	5.13	-
CuSn8Ni3Ti0.2_pt3	83.28	13.67	3.05	-
CuSn8Ni3Ti0.2_pt4	93.61	2.99	3.07	-

pt[numer], where [numer] = 1, 2, 3, 4 indicates the measurement points.

**Table 7:** Chemical composition EDS wt.% of CuSn8Ni3Ti2 alloy for the Figure 6d image.

Alloy	Chemical Composition wt.%			
	Cu	Sn	Ni	Ti
CuSn8Ni3Ti2_pt1	67.65	15.44	10.13	6.77
CuSn8Ni3Ti2_pt2	91.94	4.75	2.15	1.15
CuSn8Ni3Ti2_pt3	86.10	8.89	1.71	3.30

pt[numer], where [numer] = 1, 2, 3 indicates the measurement points.

**Table 8:** Mechanical properties of selected alloys from the Cu-Sn-Ni-Ti group.

Alloy	UTS (MPa)	Elongation A (%)	Hardness HBS
CuSn8	335	62.8	87
CuSn8Ti0.2	324	17.2	89
CuSn8Ti0.7	320	9.5	97
CuSn8Ti2	312	5.3	113
CuSn8Ni3	358	47.5	89
CuSn8Ni3Ti0.2	429	13.3	127
CuSn8Ni3Ti0.7	382	4.0	133
CuSn8Ni3Ti2	371	1.4	170

## Data Availability

The data presented in this study are available on request from corresponding author. The research results are archived in the Faculty of Foundry Engineering of the AGH University of Science and Technology in Krakow.
